# Multiple cystic swelling: Initial presentation of multiple myeloma

**DOI:** 10.4103/0971-5851.68850

**Published:** 2010

**Authors:** Sunil Kumar, A. P. Jain, Swati Waghmare

**Affiliations:** *Department of Medicine, Mahatma Gandhi Institute of Medical Sciences, Sewagram, Wardha, Maharashtra, India*

**Keywords:** *Cystic swelling*, *multiple myeloma*, *plasmacytoma*

## Abstract

Multiple myeloma, a disease allied to malignancy of reticuloendothelial cells, is not an uncommon condition. However, the diagnosis is often made quite late because the disease has multiple modes of presentation. We are reporting a case of multiple myeloma in a 55-year-old male who presented with multiple cystic swellings on the chest.

## INTRODUCTION

Multiple myeloma is a relatively rare cancer that occurs predominantly in patients over 60 years of age.[1] It is a malignant proliferation of plasma cells, primarily affecting the bone marrow and skeletal system. Occasional involvement of the extraosseous organ systems is known. Isolated cystic swelling as the initial presentation of multiple myeloma is very rare. Few case reports are available about solitary plasmacytoma and extramedullary plasmacytoma of the paranasal sinuses and soft palate.[2] We are reporting a case of multiple myeloma in a 55-year-old male who presented with multiple cystic swellings on the chest. There is no case report available.

## CASE REPORT

**Figure 1:**
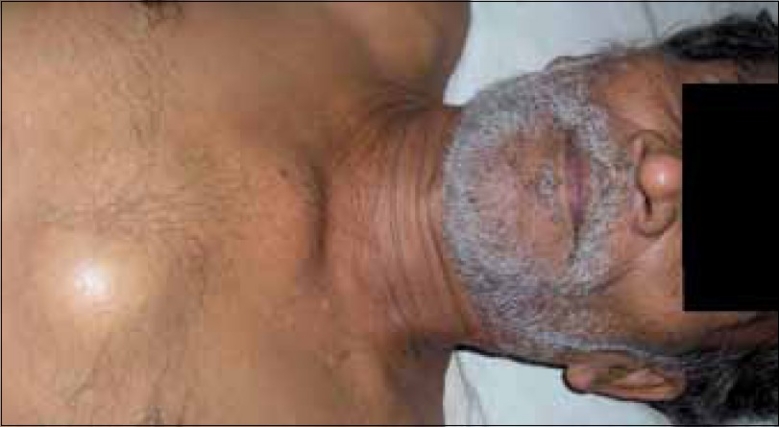
Multiple cystic swelling on the anterior chest wall

**Figure 2:**
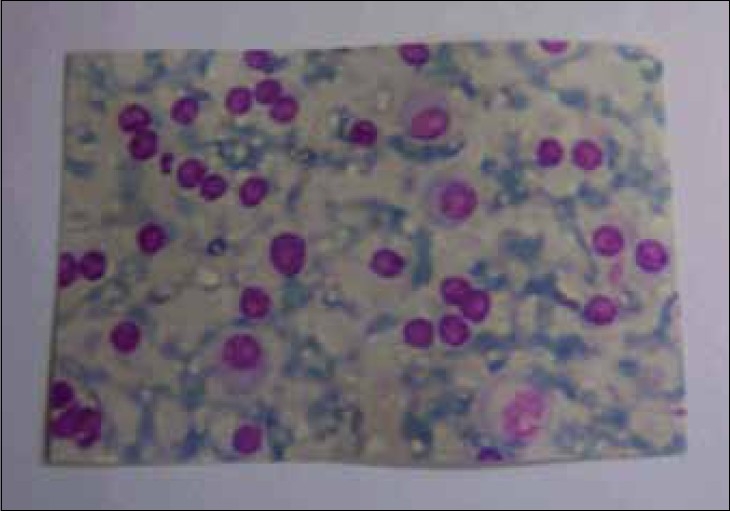
The cells bear characteristic morphologic features of plasma cells, round or oval cells with an eccentric nucleus composed of coarsely clumped chromatin and a densely basophilic cytoplasm. Binucleate and multinucleate malignant plasma cells are seen

**Figure 3:**
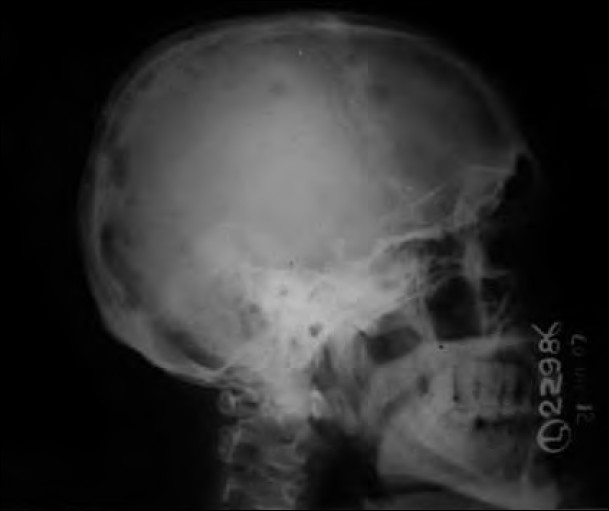
Bony lesions in multiple myeloma. The skull demonstrates the typical “punched-out” lesions characteristic of multiple myeloma

A 55-year-old male presented in the medicine outpatient department with complaints of multiple swellings on the chest wall since 2 months. There was no history of trauma, Ischemic heart disease, hypertension or diabetes. On examination, there were three cystic swellings on the anterior chest. The swelling was small to start with and increased up to 6.5 cm in 2 months [Figure 1]. It was firm, cystic and variegated in consistency and nontender on palpation. The liver was enlarged by 2 cm. Other examinations were unremarkable. His blood pressure was 130/80 mmHg. The hemoglobin was 9.6 g%, total leucocyte count was 6,300/cmm with a differential of 45% neutrophils, 37% lymphocytes, 17% monocytes and 1% eosinophils, with no myeloma cells in the peripheral smear. The erythrocyte sedimentation rate was 110 mm 1st hour (Westergren). Bence-Jones proteins was absent in the urine. Serum electophoresis shows a very thick Beta band. Serum proteins were 8.2 g%, with albumin 2.9% and globulin 4.3 g%. Blood urea and blood sugar were normal. Serum calcium, phosphorus and alkaline phosphatase were 13.2 mg%, 4.0 mg% and 7.2 Bodansky unit, respectively. Serum bilirubin was 0.3 mg%. Electrocardiogram was normal. Fine needle aspiration cytology cytology of the swelling shows plasmacytoma. The cells bear characteristic morphologic features of plasma cells, round or oval cells with an eccentric nucleus composed of coarsely clumped chromatin, and a densely basophilic cytoplasm. Binucleate and multinucleate malignant plasma cells can be seen [Figure 2]. X-ray skull showed multiple punched-out osteolytic lesions [Figure 3]. Bone marrow biopsy revealed myeloma cells and was diagnostic of multiple myeloma. The patient was started on thalidomide and corticosteroids. He also received radiotherapy for the local swelling, which was reduced on the subsequent day. He is doing well on follow-up.

## DISCUSSION

Multiple myeloma is the most common of the plasma cell neoplasms, which also includes monoclonal gammopathies of unknown significance, plasmacytomas and plasma cell leukemia. Whereas multiple myelomas represent systemic disease without the potential for cure, plasmacytomas represent local forms of plasma cell neoplasms.[3] Plasmacytoma is further classified into two groups: osseous [solitary plasmacytoma of bone (SPB)] and nonosseous [extramedullary plasmacytoma (EMP)] primary lesions.[4] EMP and SPB each comprise <4% of all plasma cell neoplasms.[5,6] Extramedullary plasmacytomas are four times more likely to occur in males than in females and 95% of the tumors occur over the age of 40 years (mean age is 59 years).[7] The majority (80%) of the EMPs occur in the in the head and neck, especially the nasopharynx and the paranasal sinuses. Rare cases of primary EMP have been described in the skull base, larynx, hypopharynx, parotid gland, submandibular gland, thyroid, mandibular region, trachea, esophagus, cervical lymph nodes, middle ear, orbit, scalp, forehead, palate, tongue and mastoid.[4–6] In our case, plasmacytoma was found as a local cystic swelling on the anterior chest wall and no other case report was found either in the literature or on the website PUBMED, although pt had other features of multiple myeloma as punched-out lesions in the skull and there was evidence of myeloma cells in the bone marrow.

The potential for malignant systemic progression is higher for solitary plasmacytomas of the bone than for extramedullary plasmacytomas.[3] Local irradiation is the primary mode of treatment for extramedullary plasmacytomas, occasionally followed by surgical resection of the residual tumor. When extramedullary plasmacytoma with multiple myeloma is diagnosed, local treatment of the plasmacytoma should be followed by the systemic combination chemotherapy.

The 5-year survival rate of extramedullary plasmacytoma is 31-75%. The prognosis of extramedullary plasmacytoma with multiple myeloma is poor and most patients die within 2 years of their diagnosis. The 3-year survival is only about 10%.[7]
